# 

**Published:** 1897-09

**Authors:** 


### ATLANTA MEDICAL AND SURGICAL JOURNAL.

VOLUME XIV NEW SERIES. ATLANTA, GA., SEPTEMBER, 1897. No. 7.

